# Effect of a 60-day weight reduction intervention prior to IVF/ICSI on perinatal outcomes in overweight or obese infertile women

**DOI:** 10.3389/fendo.2022.1062790

**Published:** 2022-12-02

**Authors:** Chen Yang, Shuheng Yang, Wei Zheng, Ruowen Zu, Shiyu Ran, Huan Wu, Bingnan Ren, Ning Lv, Yihui Kuang, Mengna Li, Jiangbo Du, Yichun Guan

**Affiliations:** ^1^ Department of Reproductive Medicine Center, The third Affiliated Hospital of Zhengzhou University, Zhengzhou, Henan, China; ^2^ Department of Nutrition, Zhejiang Nutriease Health Technology Company Limited, Hangzhou, Zhejiang, China; ^3^ State Key Laboratory of Reproductive Medicine (Henan Centre), the Third Affiliated Hospital of Zhengzhou University, Zhengzhou, Henan, China; ^4^ Department of Epidemiology, Center for Global Health, School of Public Health, Nanjing Medical University, Nanjing, Jiangsu, China; ^5^ State Key Laboratory of Reproductive Medicine, Nanjing Medical University, Nanjing, Jiangsu, China

**Keywords:** weight reduction intervention, obesity, *in vitro* fertilization, intracytoplasmic sperm injection, perinatal outcomes

## Abstract

**Purpose:**

The aim of this study was to determine whether a 60-day weight reduction intervention prior to *in vitro* fertilization/intracytoplasmic sperm injection (IVF/ICSI) and a higher weight loss ratio effectively improved perinatal outcomes for infertile overweight or obese women.

**Methods:**

This was a retrospective cohort study conducted at a university-affiliated fertility center. Two thousand three hundred and eighty-one overweight or obese infertile women who underwent or did not undergo a 60-day weight reduction intervention prior to IVF/ICSI between February 27, 2017 and November 11, 2020 were included in this study. All of these women achieved clinical pregnancy and delivered a single child after assisted reproductive technology (ART). Primary outcomes included neonatal birth weight and the incidence of pregnancy complications, premature delivery, and low birth weight.

**Results:**

The body mass index (BMI), blood glucose concentration, serum insulin level, and homeostasis model assessment of insulin resistance (HOMA-IR) of the intervention group decreased significantly after the weight reduction intervention. Neonatal birth weight was significantly higher in the intervention group (3519.6 g ±484.8 g) than the control group (3406.8 g ± 554.2 g; *P* < 0.001). There was no significant difference in the incidence of pregnancy complications between the two groups. Linear regression analysis found that the weight reduction intervention prior to IVF/ICSI and lower HOMA-IR at ovulation induction were associated with increased birth weight. As the weight loss ratio increased, the incidence of hypertensive disorders of pregnancy, premature membrane rupture, premature delivery, stillbirth, and low birth weight showed a downward trend.

**Conclusion(s):**

A 60-day weight reduction intervention prior to IVF/ICSI may increase neonatal birth weight, reduce maternal blood glucose concentration, and improve maternal insulin resistance in infertile overweight or obese women. This results require to be further verified by prospective randomized controlled trials with a larger sample size.

## Introduction

Fueled by rapid social development, an increasingly sedentary lifestyle, and a nutritional transition to processed foods and high-calorie diets in recent years, the incidence of overweight and obesity expanded rapidly and the number of cases of infertility caused by overweight and obesity is also increasing (1, 2). For adults, the world health organization (WHO) defined body mass index (BMI) ≥ 25 kg/m^2^ as overweight and BMI ≥ 30 kg/m^2^ as obesity (2). Overweight and obesity increases the risk of infertility by causing functional alterations to the hypothalamic–pituitary–ovarian axis (3). Overweight and obesity also impairs ovarian responsiveness to gonadotrophin stimulation, such that higher doses of medication are required. Moreover, compared with women of normal weight, obese women have an increased risk of cycle cancellation and decreased risk of implantation, clinical pregnancy and live birth (4, 5). Overweight and obesity are associated with lower live birth rates in women undergoing assisted reproductive therapy (ART), and previous studies have demonstrated that weight loss prior to ART in overweight and/or obese women increases pregnancy and live birth rates (6–10). Meanwhile, obesity increases the risk of premature delivery, low birth weight, miscarriage, abnormal labor and postpartum complications (11–15). Therefore, a weight reduction intervention prior to *in vitro* fertilization/intracytoplasmic sperm injection (IVF/ICSI) may be successful at improving ART and perinatal outcomes and may be an appropriate treatment for patients with obesity-related endocrine disorders and irregular menstruation (16). However, there is a lack of researches on whether weight reduction prior to IVF/ICSI affects perinatal outcomes. In addition, there is an urgent need to determine the range of weight loss ratios that is most likely to improve perinatal outcomes. Information gained through studying the effects of weight reduction and the optimal weight loss ratio on perinatal outcomes is required to ensure that overweight and obese infertile patients who accept a weight reduction intervention prior to IVF/ICSI have nicer perinatal outcomes.

## Materials and methods

### Study design and patients

This was a retrospective cohort study performed at the Assisted Reproduction Center of the Third Affiliated Hospital of Zhengzhou University. Two thousand three hundred and eighty-one overweight or obese infertile women who voluntarily underwent (intervention group, n=495) or rejected/discontinued a 60-day weight reduction intervention (control group, n=1,886) prior to IVF/ICSI from February 27, 2017 to November 11, 2020 and the 2,381 singletons delivered by these patients were included. Patients were randomly selected from the medical record system. The weight reduction intervention was defined as a 60-day weight reduction program that included a low-carbohydrate diet, diet structure adjustment, and an exercise program under the guidance of nutritionists. Nutritionists in the weight management department of the reproductive center instructed patients with a high BMI to purchase appropriate foods and consume a low-carbohydrate diet, to choose foods with a low glycemic index to induce fat breakdown, and to exercise appropriately so that their energy expenditure exceeded their intake. The inclusion criteria of the intervention group included: i) accepting the weight reduction intervention prior to IVF/ICSI, ii) a BMI ≥ 25 kg/m^2^, iii) undergoing IVF/ICSI after the weight-loss intervention, and iv) delivering a single child. The inclusion criteria for the control group differed in that these patients either interrupted or rejected the weight reduction intervention. Patients were excluded from the study if they had a diagnosis of recurrent spontaneous abortion, repeated implantation failure, abnormal anatomical structure of the uterus, chromosomal abnormalities, or tumors. This study was approved by the Ethics Committee of the Third Affiliated Hospital of Zhengzhou University (2021-077-01). Nutritionists supervised and recorded the implementation of weight reduction by providing substitute meals, telephone follow-up, and wechat interaction, guided and adjusted according to the patients’ feedback, and recorded various physical function indexes after weight reduction. All patients included in the study were pregnant after IVF/ICSI and delivered successfully. This study included embryo transfer (ET) and frozen-thawed embryo transfer (FET) cycles.

### Controlled ovarian hyperstimulation, endometrial preparation, and embryo transfer

Based on the patients’ condition, a controlled ovarian hyperstimulation protocol (gonadotropin releasing hormone agonist (GnRH-a) long-protocols or GnRH-antagonist protocols) was formulated and follicular growth was monitored by transvaginal ultrasound. The specific methods of ovarian stimulation were described in previous publications by researchers at our center (17). When the diameter of at least one dominant follicle was more than 20 mm, or the diameters of three follicles were more than 18 mm, the ovarian hyperstimulation program was stopped and recombinant human chorionic gonadotropin (Ezer, Merck, Germany) was used to trigger ovulation. Thirty-six hours later, the oocytes were removed under transvaginal ultrasound monitoring. Routine luteal support was given after oocyte retrieval, and IVF or ICSI was performed depending on the patients’ condition. Fresh embryo transfer was performed 3 days after oocyte retrieval or with blastocyst transplantation 5 days after oocyte retrieval. An endometrial preparation protocol for frozen-thawed embryo transfer, including natural cycles, artificial cycles, GnRH-a pre-treatment in hormone replacement therapy (HRT), and induced ovulation cycles, was devised based on the patients’ condition. Natural cycles were applied to patients with regular menstruation, and they usually induces ovulation with or without minimum human chorionic gonadotropin (HCG) that results in the least risk of adverse effects and cost. For convenience, patients with or without normal menstrual cycles all can be treated with artificial cycles which were performed using estrogen and progesterone (18). Artificial cycles can optimize endometrial receptivity with a more consistent interval, duration, and concentration of estrogen and progesterone compared to natural cycles. GnRH-a pre-treatment in HRT cycles were commonly used in patients with polycystic ovary syndrome to downregulate any hormones produced by the ovaries in subsequent therapy when using HRTs (19). Induced ovulation cycles were applied to patients with abnormal ovulation. Either cleavage-stage embryos were transferred on the third day after ovulation or blastocysts were transferred on the fifth day after ovulation. Vaginal ultrasound was used to monitor the presence of a gestational sac, an embryo, and a fetal heart rate on day 30 after transplantation. Basic clinical data and hormone levels were obtained from the case system. Patients were followed up with telephone calls until the end of their pregnancy, and any pregnancy complications and neonatal outcomes were obtained from the medical record system.

### Outcome measures

Data related to pregnancy complications and neonatal outcomes were obtained from a review of the patients’ medical charts or through follow-up telephone calls. Hypertensive disorders of pregnancy include the following four categories: chronic hypertension, gestational hypertension, preeclampsia–eclampsia, and chronic hypertension with superimposed preeclampsia (20, 21). Chronic hypertension during pregnancy is defined as the presence of hypertension before conception, the development of elevated blood pressure before a gestational age of 20 weeks, or the persistence of hypertension beyond 12 weeks after delivery. Gestational hypertension, formerly known as pregnancy-induced hypertension, is defined as the development of essential hypertension after 20 weeks of gestation, in the absence of diagnostic criteria for preeclampsia. Preeclampsia is defined as new-onset hypertension that develops after 20 weeks of gestation and is accompanied by proteinuria or evidence of end-organ dysfunction. Eclampsia is defined as seizures in a pregnant woman with preeclampsia with no other identifiable cause. Gestational diabetes, defined as diabetes diagnosed during pregnancy, is a condition in which women without a previous diagnosis of diabetes exhibit abnormal blood glucose concentrations during pregnancy (22, 23). The incidences of placenta praevia (24), premature membrane rupture (25), and stillbirth (26) were also determined. A live birth was defined as an infant born alive after at least 28 weeks of gestation who survived more than 28 days, and a postterm pregnancy was defined as a gestational age > 42 weeks (27, 28). The WHO defines preterm birth as birth before 37 gestational weeks. Birth weights < 2500 g were classified as low birth weight, and birth weights < 1500 g were classified as very low birth weight (27, 29).

### Statistical analysis

Continuous variables are presented as means ± standard deviations and categorical variables are presented as frequencies and corresponding percentages. The differences between two groups were assessed using a Student’s *t-*test for continuous variables and a Pearson’s chi-square test or Fisher’s exact probability method for categorical variables. Logistic and linear regression analyses were used to assess perinatal outcomes. Linear regression analysis was used to identify the factors influencing neonatal birth weight. All statistical analyses were performed using SPSS 22.0 software (IBM, Armonk, NY, USA). Two-tailed *P* values < 0.05 were considered statistically significant.

## Results

### Characteristics of the intervention group and the control group

Four hundred and ninety-five singletons from mothers who underwent a weight reduction intervention prior to IVF/ICSI and 1,886 singletons from mothers who directly entered the ovulation induction cycle without a weight reduction intervention were included in this study. The basic characteristics of the intervention group and the control group are presented in **Table 1**. Anti-Müllerian hormone (AMH) concentration and antral follicle count (AFC) were significantly higher in the intervention group than the control group. There were no significant differences in age; infertility duration; cause of infertility; embryo transfer period; endometrial thickness on the day of embryo transfer; basal follicle-stimulating hormone (bFSH) dose; or free triiodothyronine (FT3), free thyroxine (FT4), and thyroid-stimulating hormone (TSH) concentrations between the two groups.

**Table 1:** Characteristics of the intervention group and the control group.

Characteristics	Intervention group	Control group	*P* value
Singleton, n	495	1886	
Age (y)	30.2 ± 4.9	30.5 ± 4.4	0.447
Infertility duration (y)	4.0 ± 3.3	3.7 ± 3.0	0.367
Causes of infertility			
Polycystic ovary syndrome	178 (36.0%)	639 (33.9%)	0.386
Pelvic and tubal factors	235 (47.5%)	955 (50.6%)	0.211
Hypoovarianism	45 (9.1%)	223 (11.8%)	0.087
Male factor	195 (39.4%)	697 (36.9%)	0.319
Other	48 (9.7%)	199 (10.6%)	0.579
Embryo transfer period			
Day 3	203 (41.0%)	845 (44.8%)	0.130
Day 5/6	292 (59.0%)	1041 (55.2%)	
Endometrial thickness at that day on embryo transfer (mm)	10.5 ± 2.0	10.4 ± 2.7	0.409
AMH(pmol/L)	31.5 ± 23.4	24.2 ± 22.3	<0.001
AFC	20.3 ± 7.6	18.2 ± 7.5	<0.001
bFSH (IU/L)	7.0 ± 4.1	6.6 ± 4.0	0.281
FT3 (pmol/L)	5.6 ± 0.7	5.0 ± 0.6	0.118
FT4 (pmol/L)	17.2 ± 1.9	16.9 ± 1.8	0.373
TSH (mIU/L)	2.1 ± 0.9	2.7 ± 0.9	0.122

Mean + SD/N(%).

SD, standard deviation; AMH, anti-Müllerian hormone; AFC, antral follicle count; bFSH, basal follicle stimulating hormone; FT3, free triiodothyronine; FT4, free thyroxine; TSH, thyroid stimulating hormone.

### Comparison of blood glucose and insulin levels between two groups with the change of BMI

Insulin resistance test and oral glucose tolerance test (OGTT) results were compared after weight loss (**Table 2**). The BMI of the intervention group decreased significantly after the intervention (*P* < 0.001), while the BMI of the control group decreased slightly. Fasting glucose, 1-hour blood glucose, fasting insulin, 1-hour insulin, 2-hour insulin concentrations, and HOMA-IR values were lower after the intervention than before the intervention. The OGTT and insulin resistance test results did not change significantly in the control group after the change in BMI.

**Table 2:** Comparison of blood glucose and insulin levels between two groups with the change of BMI.

Item	Intervention group		*P* value	Control group		*P* value
	Initial	At ovulation induction		Initial	At ovulation induction	
BMI (kg/m²)	30.9 ± 4.9	27.7 ± 4.5	<0.001	30.3 ± 3.7	29.9 ± 3.8	0.485
Glu0 (mmol/L)	6.0 ± 1.6	5.3 ± 1.3	0.045	5.9 ± 1.5	5.8 ± 1.5	0.702
Glu1 (mmol/L)	9.9 ± 2.4	9.0 ± 1.8	0.003	9.7 ± 3.1	9.7 ± 2.9	0.909
Glu2 (mmol/L)	8.7 ± 1.9	8.4 ± 1.8	0.118	7.3 ± 2.5	7.1 ± 1.4	0.511
INS0 (μU/mL)	20.6 ± 4.0	12.8 ± 3.1	<0.001	19.7 ± 4.1	18.2 ± 3.4	0.244
INS1 (μU/mL)	100.9 ± 34.9	86.8 ± 41.0	<0.001	96.5 ± 27.9	94.9 ± 23.2	0.132
INS2 (μU/mL)	90.2 ± 26.4	83.2 ± 22.8	0.004	87.0 ± 17.4	86.6 ± 15.4	0.493
HOMA-IR	3.5 ± 1.8	2.7 ± 1.3	<0.001	3.3 ± 1.7	3.3 ± 0.9	0.855

Mean + SD.

SD, standard deviation; Glu0, fasting blood glucose level; Glu1, 1-hour blood glucose level; Glu2, 2-hour blood glucose level; INS0, fasting insulin level; INS1, 1-hour insulin level; INS2, 2-hour insulin level; HOMA-IR, homeostasis model assessment of insulin resistance, (Glu0*INS0)/22.5.

### Comparison of perinatal outcomes of the intervention group and the control group

The comparison of perinatal outcomes between the the intervention group and the control group after adjusting for confounding factors (age, infertility duration, AMH concentration, AFC) is shown in **Table 3**. The incidences of hypertensive disorders of pregnancy, gestational diabetes, placenta praevia, premature membrane rupture, premature delivery, postterm delivery, stillbirth, low birth weight, very low birth weight, birth defect, and neonatal asphyxia were not significantly different between two groups. Birth weight was significantly higher in the intervention group (3519.6 ± 484.8) g than the control group (3406.8 ± 554.2) g after adjusting for confounding factors (*P* < 0.001).

**Table 3:** Comparison of perinatal outcomes of the intervention group and the control group.

Perinatal outcomes	Intervention group	Control group	Adjusted OR/MD(95% CI)	Adjusted *P* value
Live births, n	493	1877		
Hypertensive disorders of pregnancy (rate %)^a^	38 (7.7%)	178 (9.4%)	0.785 (0.537,1.149)	0.214
Gestational diabetes (rate %)^a^	30 (6.1%)	155 (8.2%)	0.695 (0.455,1.061)	0.092
placenta praevia (rate %)^a^	3 (0.6%)	10 (0.5%)	1.112 (0.290,4.266)	0.877
Premature rupture of membranes (rate %)^a^	15 (3.0%)	74 (3.9%)	0.786 (0.439,1.409)	0.419
Premature delivery (rate %)^a^	35 (7.1%)	192 (10.2%)	0.804 (0.559,1.156)	0.239
Postterm delivery (rate %)^a^	0	9 (0.5%)	\	\
Stillbirth (rate %)^a^	2 (0.4%)	9 (0.5%)	0.749 (0.156,3.596)	0.718
Birth weight (g)	3519.6 ± 484.8	3406.8 ± 554.2	106.938 (53.022,160.853)	<0.001
Low birth weight (rate %)^b^	41 (8.3%)	176 (9.3%)	0.875 (0.604,1.268)	0.481
Very low birth weight (rate %)^b^	4 (0.8%)	18 (1.0%)	0.742 (0.242,2.279)	0.603
Birth defects (rate %)^b^	0	9 (0.5%)	\	\
Neonatal asphyxia (rate %)^b^	0	4 (0.2%)	\	\

Mean + SD/N(%).

SD, standard deviation; OR, odds ratio; MD, mean deviation; CI, confidence interval.

^a^The denominator was the number of clinical delivery patients; ^b^The denominator was the number of live births.

Adjusted factors included age, infertility duration, AMH concentration, AFC.

### Linear regression analysis of the influencing factors of birth weight

As shown in **Table 4**, a linear regression analysis found that a weight reduction intervention prior to IVF/ICSI and lower HOMA-IR at ovulation induction were associated with increased neonatal birth weight. Maternal age, infertility duration, endometrial thickness at that day on embryo transfer, AMH concentration, AFC, bFSH dose, TSH concentration and initial HOMA-IR had no effect on birth weight.

**Table 4:** Linear regression analysis of the influencing factors of birth weight.

Item	MD (95% CI)	*P* value
Weight reduction intervention prior to IVF/ICSI	104.794 (50.171~159.418)	<0.001
Maternal age (years)	4.118 (-1.121~9.356)	0.123
Infertility duration (years)	-3.678 (-10.979~3.622)	0.323
Endometrial thickness at that day on embryo transfer (mm)	5.042 (-3.283,13.367)	0.235
AMH (pmol/L)	-0.007 (-1.263~1.249)	0.991
AFC (n)	-0.916 (-2.168~0.337)	0.152
bFSH (IU/L)	0.969 (-3.643~5.581)	0.680
TSH (mIU/L)	2.280 (-0.191~4.752)	0.071
Initial HOMA-IR	2.668 (-23.262~28.599)	0.840
HOMA-IR at ovulation induction	-31.419 (-58.023~-4.814)	0.021

MD, mean deviation; CI, confidence interval; IVF, *in vitro* fertilization; ICSI, intracytoplasmic sperm injection; AMH, anti-Müllerian hormone; AFC, antral follicle count; bFSH, basal follicle stimulating hormone; TSH, thyroid stimulating hormone; HOMA-IR, homeostasis model assessment of insulin resistance.

### Comparison of the incidence of perinatal outcomes with different weight loss ratio

**Figure 1:**
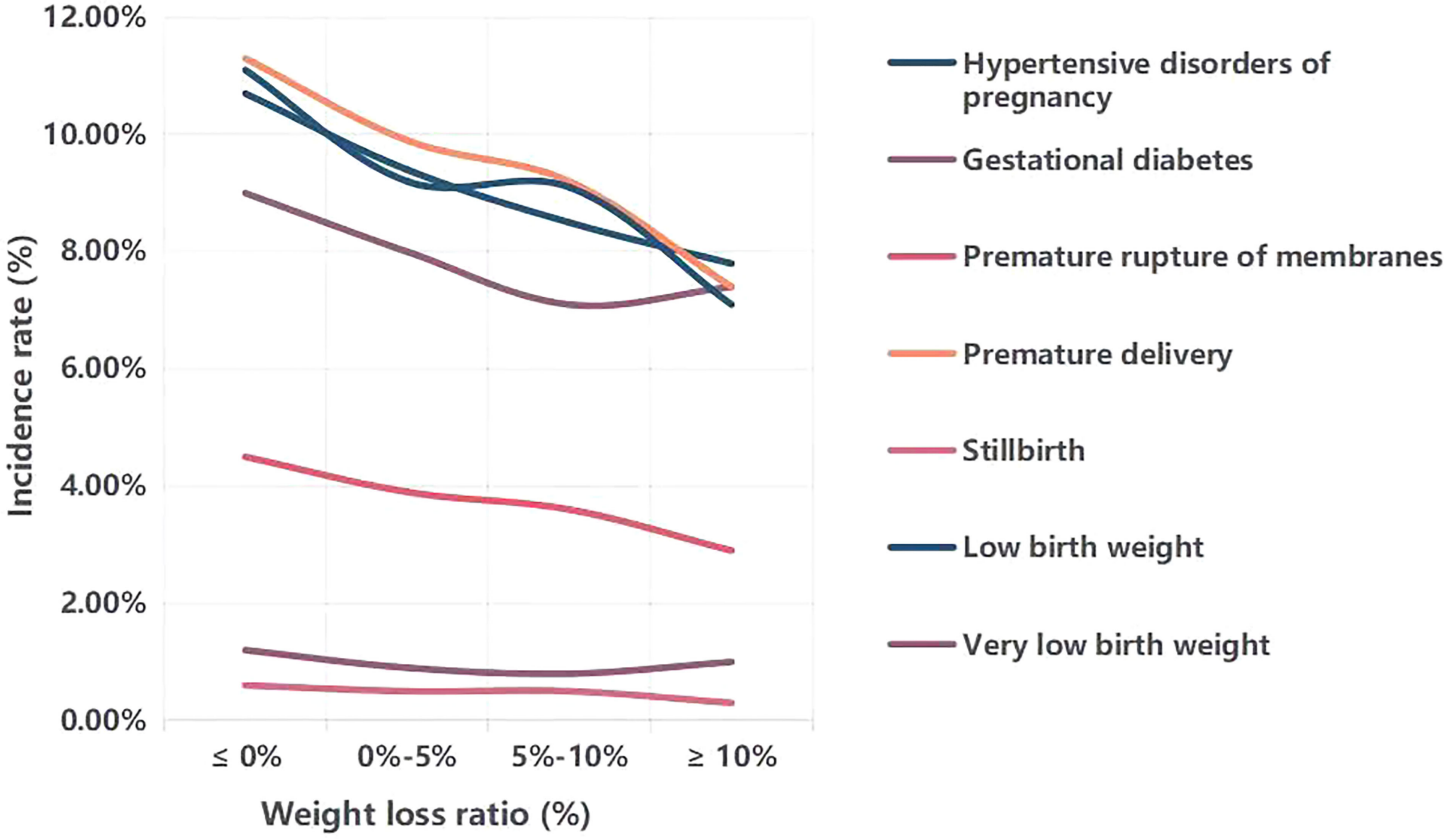
Comparison of the incidence of perinatal outcomes with different weight loss ratio. Weight loss ratio, (initial weight-weight at ovulation induction)/initial weight*100%.

**Figure 1** demonstrates the influence of different weight loss ratios on perinatal outcomes. We divided the entire cohort into four weight loss ratio groups (≤0%, 0%-5%, 5%-10%, and ≥10%). With an increase in the weight loss ratio, the incidences of hypertensive disorders of pregnancy, premature membrane rupture, premature delivery, stillbirth and low birth weight gradually decreased. However, the incidences of gestational diabetes and very low birth weight reached their lowest levels at a weight loss ratio of 5%-10%.

## Discussion

Compared with women with a normal BMI, overweight and obese women have a greater risk of adverse pregnancy outcomes, including preeclampsia, gestational diabetes, stillbirth, and cesarean section (30, 31). A meta-analysis confirmed the increased risk of fetal birth defects (neural tube defects, spina bifida, cardiovascular abnormalities, ventricular septal abnormalities, cleft palate, cleft lip and palate, anorectal malformations, hydrocephalus, and limb reduction) in children born to obese mothers (32). This may be related to maternal metabolic disorders (33). Overweight and obesity are also well-described risk factors for infertility (3). Therefore, identifying an appropriate weight loss ratio and determining whether weight loss before ovulation induction is beneficial to perinatal outcomes are topics of widespread concern. To the best of our knowledge, weight reduction in obese women scheduled for IVF/ICSI does not increase the incidence of live births (34, 35) or the cumulative live birth rate (36). However, Liu et al. (37) reported that an appropriate amount of weight loss in overweight or obese women is associated with a significant reduction in the incidence of perinatal complications. Our study focused on the effect of a weight reduction intervention for overweight or obese women prior to IVF/ICSI on perinatal outcomes.

We found that a weight-loss intervention prior to IVF/ICSI increased neonatal birth weight, and this may be related to improvements in placental blood flow by reducing insulin resistance. In a large randomized controlled trial, 290 women were assigned to a 6-month lifestyle-intervention program prior to 18 months of infertility treatment (intervention group) and 290 women were assigned to immediate infertility treatment for 24 months (control group) (38). Birth weight was higher in the intervention group than the control group, which is consistent with the results of our study. In our study, there were no differences in the incidence of pregnancy complications, including hypertensive disorders of pregnancy, gestational diabetes, placenta praevia, and premature membrane rupture, between the intervention and control groups. These are similar to the findings of Mutsaerts and Chavarro (6, 38). In a retrospective cohort study, overweight or obese infertile women who achieved a weight loss ratio > 10% had higher conception rates and live birth rates (39), indicating that perinatal outcomes may be improved with an increased weight reduction ratio. It is worth noting that excessive weight loss over a short time is discouraged, as it has been reported to adversely affect ART outcomes (40) and is associated with an increased risk of adverse pregnancy outcomes, such as low birth weight and miscarriage (41). Overall, weight reduction prior to ART is a reliable treatment method for overweight and obese women preparing for pregnancy, and the inclusion of weight reduction in the pre-pregnancy treatment regime of obese infertile patients is recommended (42).

This study is a retrospective cohort study with limited sample size. As some patients were followed up by telephone, recall bias may have been introduced. This study also enhanced the weight management awareness of patients in the intervention group during assisted reproductive treatment and pregnancy stage, but a handful of patients might give up weight control midway during the whole pregnancy process which affected the results of our study. Nevertheless, compared with previous similar studies, the perinatal outcomes of this study were more comprehensive and the trend of perinatal outcome incidence rate with the change of weight loss ratio was explored for the first time. In addition, there are few studies on the influence of weight reduction in overweight and obese infertile women receiving IVF/ICSI treatment on perinatal outcomes, and there are no guidelines for optimal weight loss rates. Therefore, this study provides significant references to clinicians and overweight or obese patients. Meanwhile, more studies and larger sample sizes are needed to verify the results of this study and to draw more comprehensive and objective conclusions.

## Conclusion

In conclusion, weight reduction intervention prior to IVF/ICSI may increase neonatal birth weight, reduce maternal blood glucose concentration, and improve maternal insulin resistance in infertile overweight or obese women. The present study may therefore be helpful for patients with excessive BMI to receive a weight-loss intervention when attending weight counseling before initiating IVF/ICSI cycles.

## Data availability statement

The original contributions presented in the study are included in the article/supplementary material. Further inquiries can be directed to the corresponding authors.

## Ethics statement

This study was approved by the Ethics Committee of the Third Affiliated Hospital of Zhengzhou University (ethics approval number: 2021-077-01).

## Author contributions

CY: writing the article, study conception and design, and critical review of the article. SY, WZ, RZ, SR, HW, BR, NL, YK and ML: data processing. JD and YG: supervising the study. All authors contributed to the article and approved the submitted version.

## Funding

This study was funded by State Key Laboratory of Reproductive Medicine, Nanjing Medical University (SKLRM-K201902), National Key R&D Program “Fertility Health and Health Security for Women and Children” (2021YFC2700602), Henan Young and Middle-aged Health Science and Technology Innovation Leading Talent Training Project (No. YXKC2021020) and National Health Commission Scientific Research Foundation Henan Medical Science and Technology Research Program Provincial Joint Construction Project (No. SBGJ202102180).

## Acknowledgments

Thanks to Professor Guan Yichun and other authors for their support and help.

## Conflict of interest

Author NL was employed by company Zhejiang Nutriease Health Technology Company Limited.

The remaining authors declare that the research was conducted in the absence of any commercial or financial relationships that could be construed as a potential conflict of interest.

## Publisher’s note

All claims expressed in this article are solely those of the authors and do not necessarily represent those of their affiliated organizations, or those of the publisher, the editors and the reviewers. Any product that may be evaluated in this article, or claim that may be made by its manufacturer, is not guaranteed or endorsed by the publisher.
